# The combined effects of motor imagery and motor practice are influenced by differences in working memory function: Examination of brain, spinal cord, and muscle performance data

**DOI:** 10.1016/j.ibneur.2025.06.016

**Published:** 2025-06-25

**Authors:** Yuki Fukumoto, Hiroki Bizen, Marina Todo, Naoki Yoshida, Toshiaki Suzuki

**Affiliations:** a Kansai University of Health Sciences, Faculty of Health Sciences, Department of Physical Therapy, 2-11-1 Wakaba Sennangun Kumatori, Osaka 590-0482, Japan; b Graduate School of Kansai University of Health Sciences, Graduate School of Health Sciences, 2-11-1 Wakaba Sennangun Kumatori, Osaka 590-0482, Japan; c Kansai University of Health Sciences, Faculty of Health Sciences, Department of Occupational Therapy, 2-11-1 Wakaba Sennangun Kumatori, Osaka 590-0482, Japan

**Keywords:** Motor imagery, Motor learning, F-waves, Oxy-hb, Finger dexterity

## Abstract

The ability to perform motor imagery is affected by differences in short-term memory storage capacity in terms of the activation of working memory. Therefore, from the viewpoint of the simultaneous measurement of brain activation and spinal motor neuron excitability, we examined differences in the combined effects of motor practice and motor imagery due to differences in working memory function. 20 healthy individuals were classified into Normal (score of ≤5–6 digits) and Good (score of ≥7 digits) groups based on working memory in a digit span test. Following this, participants performed six sets of repetitive exercises combining motor practice and motor imagery, and changes in neural activity patterns in the brain and spinal cord, as well as changes in finger dexterity, were tracked. In brain network analysis, the first execution of the imagery showed high Betweenness Centrality in the frontal pole cortex, which shifted to the dorsolateral prefrontal cortex with repeated imagery. The involvement of the frontal pole may reflect introspection of motor behavior in the initial stage, while the dorsolateral prefrontal cortex consistently participated in imagery generation throughout the entire motor imagery process. In addition, both groups showed improvement in finger dexterity after intervention, but during repetitive motor imagery, a decrease in amplitude F/M ratio was observed in the Good Working Memory group, and a decrease in activation of the right primary motor cortex was observed in the Normal Working Memory group. In terms of working memory, especially in aspects of the phonological loop, those with higher function may execute motor imagery more distinctly.

## Highlights

• Working memory differences involve central nervous function during motor imagery.
• High working memory lowers spinal motor neuron excitability during motor imagery.
• Low working memory lowers activity in the primary motor cortex during motor imagery.
• Differences in working memory do not affect motor skills after motor imagery.
• Phonological loop is related to the quality of motor imagery.

## 1 Introduction

When motor skills are impaired due to some disease, it is common to relearn these skills through the repetition of actual execution (AE) accompanied by motor execution. However, repetitions of AE show a ceiling effect and the fast-learning effect per repetition is limited (Miyaguchi et al., 2019). The usefulness of repetition in motor learning is related to the intertrial interval that exists between repeated AEs (Bilodeau and Bilodeau, 1958). During this period, the motor program is modified as needed, considering errors from the memory of the previous AE (Salmoni et al., 1984). Such cognitive activities are similar to the definition of motor imagery practice (MIP) in terms of motor memory reproduction; MIP is the reproduction of memory, defined as the activation of cognitive processes using working memory (WM) (Fara, 1989). Recently, it was shown that motor learning may be promoted by MIP alone, even without motor execution (Kim et al., 2022, Ruffino et al., 2019). Therefore, a combined treatment strategy of AE and MIP (AEMIP) is considered to be a useful tool for motor learning because it leads to the simultaneous reinforcement of high-level circuits due to overlapping bottom-up and top-down effects (Sheahan et al., 2018, Bellelli et al., 2010). Although the acquisition of finger dexterity is considered insufficient in stroke patients compared to the activity of the lower limbs (Langhorne et al., 2009), AEMIP improves upper limb dysfunction (Page et al., 2001) and hand function (Liu et al., 2014) compared to AE alone.

However, a few reports have suggested that AEMIP is not particularly effective in some cases (Bovend’Eerdt et al., 2009, Casby and Moran, 1998). This discrepancy in results is because the procedures for the implementation MIP are not standardized (Schuster et al., 2011). Therefore, we set out to address this methodological concern. First, we clarified that, as an effect of implementing MIP alone, by having participants recall a composite of information related to a motor task (information indirectly associated with muscular sensation) in addition to kinesthetic MIP, MIP can be performed clearly and an improvement in finger dexterity can be expected (Fukumoto et al., 2022a). Expanding on this effort, we attempted to correct methodological concerns from the perspective of MIP strategies, and found that six sets of repetitions of AEMIP could be expected to improve finger dexterity significantly more than six sets of repetitions of AE alone (Fukumoto et al., 2024a). However, the effect of AEMIP was not greater than that of AE alone in all subjects, and a problem of so-called “individual differences” was observed.

When motor skill improvement is observed with AEMIP, it is clear that neurophysiological changes are involved, since muscle hypertrophy and the like may not occur immediately. Therefore, it is important to examine the neurophysiological background of the AEMIP effect, which should be judged initially from the changes in motor skills, and secondly from the neurophysiological background. There is a certain consensus that the activation of central brain regions involved in motor execution, mainly in the supplementary motor area (SMA), is involved in the functional changes observed in the brain during MIP (Lotze et al., 1999, Nakano et al., 2012, Roland et al., 1980). Furthermore, the possibility that central brain regions centering on the SMA influence excitatory changes in spinal motor neuron function has also been clarified (Fukumoto et al., 2021, Fukumoto et al., 2022b). Conversely, although the motor aspects of the spinal cord are involved in finger dexterity (Fukumoto et al., 2023), contradictory results have been shown regarding the changes in spinal motor neuron excitability during MIP, with a number of reports finding no change in excitability (Patuzzo et al., 2003, Liepert and Neveling, 2009), and some reporting an increase (Suzuki et al., 2014, Rossini et al., 1999). Such discrepancies may be related to individual differences in WM function. In the multi-component model of WM, one central executive is supposed to integrate information and manage the slave system, and there are three slave systems under its supervision. visuo- Within the spatial sketch pad, Inner Scribe, which is the active system involved in the maintenance of spatial information such as position and series and in the planning of body movements, manipulates and processes images through functions involved in the planning of visual movements, it can be taken that MIP would be the result of the action of the central executive on the content temporarily held in the visuo-spatial sketch pad. Recently, however, it has become clear that the phonological loop can also generate individual differences in the effects of MIP, since MIP is often executed under verbal instructions, although it does not serve as the original memory for the generation of MIP (Fukumoto et al., 2024b). Specifically, in the digit span for estimating the phonological loop, it seems that MIP cannot be performed well when the cutoff value is less than 4 digits (2–4 digits), but when the cutoff value is greater than 5 digits (5–9 digits), it is doubtful whether the same MIP effect can be expected regardless of whether the performance is high or low. In particular, short-term memory storage capacity, such as the phonological loop, increases with age, but large developmental and individual differences are found even within the same age group (Gathercole et al., 2003). Given that MIP activates WM, poor WM function makes it difficult to execute clear MIP and generates individual differences in the ability to perform MIP. The clarity of MIP is also closely related to the activation of the neural substrate, and it has been shown that the primary motor area (M1) is less likely to be activated when clear MIP is not performed (Dechent et al., 2004), while increased excitability is less likely to be observed when MIP execution is clear from the perspective of spinal motor neuron excitability (Nomura et al., 2017).

Therefore, if a certain level of WM function is ensured, the MIP effect can be relatively enjoyed, but the clarity of the MIP being performed should be different, so the state of activity of the central nervous system base should be different for both. In other words, we hypothesized that, although motor skill improvement by MIP can be expected if a certain level of WM function is secured, the higher the WM function, the less need to increase spinal motor neuron excitability, while with relatively lower WM function, the harder it is to obtain activation of the M1. On the basis of these hypotheses, we examined whether differences in the effects of AEMIP occur due to differences in WM function, even if WM function exceeds a certain level, based on the simultaneous measurement of brain activation and spinal motor neuron excitability in addition to motor skill changes.

## 2 Methods

### 2.1 Participants

Because disease characteristics are expected to interfere with experimental results, healthy college students were included in this study. Participants had no previous experience participating in MIP-related research, had not undergone WM assessment, had not been evaluated for finger dexterity using the methods employed in this study, and were novices in all respects. The exclusion criteria were subjects who did not possess WM function above a certain level, specifically those who had a score of ≤ 4 digits in the Digit span: Backward test (Fukumoto et al., 2024b).

G*power software (ver. 3.1.9.4; Heinrich Heine University, Dusseldorf, Germany) was used for the analysis of variance in repeated measures, within-between interactions. The type of power analysis was set to “a priori: compute required sample size- given α, power, and effect size.” The effect size f was set to 0.4, α error probability was set to 0.05, power (1-β error probability) was set to 0.8, number of groups = 4, number of measurements = 4, correlation among repetitive measures was set to 0.5, and nonsphericity correction epsilon was set to 1. The sample size was calculated to be 12. A certain number of subject dropouts were also expected, and 21 healthy right-handed adults were recruited from self-declaration. The subjects of this study were fully informed of the significance and purpose of the study and their written consent was obtained for the measurements in accordance with the Declaration of Helsinki (Kansai University of Health Sciences Research Ethics Review Committee, Ethics No.: 23–20).

### 2.2 Experimental setup

The experiment consisted of an evaluation of WM function, motor skill improvement by AEMIP, and changes in brain activation and spinal motor neuron excitability.

### 2.3 Evaluation of WM function

The capacity of short-term memory storage, especially the phonological loop that stores verbalized information, can be assessed by Digit Span, a widely used subtest of the Clinical Assessment of Attention. Two series of numbers ranging from two to nine digits were presented verbally in ascending order, with the number of digits increasing. The first series of numbers was presented verbally, but in case of an incorrect answer, the number was moved to the second series of the same digits. Individual differences in the effect of MIP performed under verbal instructions may be influenced by the capacity-constraining nature of the phonological loop (Fukumoto et al., 2024b). Therefore, following previous studies, we employed the backward method, which is considered to reflect WM function in particular, from the viewpoint that the temporary retention of information and the task of “saying it backward” proceed simultaneously (Carlson et al., 2002). Furthermore, in this study, to clarify the MIP effect brought about by cognitive functions derived from Digit Span: Backward, it is necessary to capture differences in physical factors directly contributing to the learning process and minimize the influence of individual differences. While it has been suggested that the MIP effect can be achieved even with low initial motor skills through appropriate guidance and practice (Schuster et al., 2011), it has also been reported that individuals with low motor skills are less likely to benefit from the MIP effect (Mao et al., 2022, Dahm and Rieger, 2019, Kraeutner et al., 2018). Therefore, we also confirmed whether there were any differences in initial motor skills between the two groups divided based on WM.

### 2.4 Verification of the effectiveness of AEMIP

**Fig. 1:**
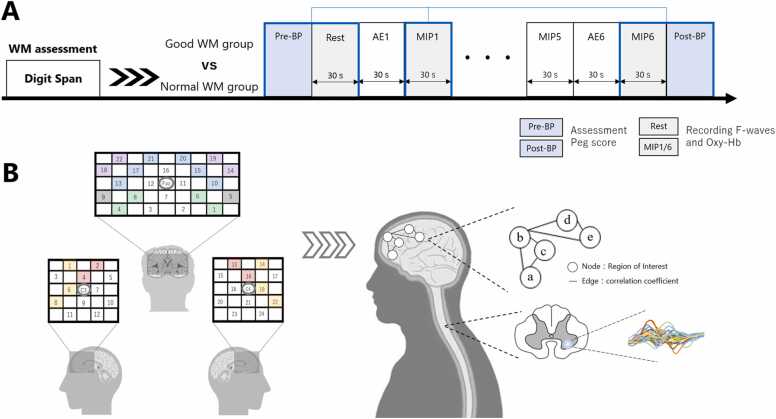
Flow of the study and evaluation of the neural basis. (A) The flow of the study, in which scores were assigned to the Good Working Memory (WM) group and the Normal WM group according to the number of digits that could be recited backward. Both groups underwent the same research process. Six sets of a combination of actual execution (AE) and motor imagery practice (MIP) were repeated, and changes in finger dexterity were evaluated before and after this intervention. Blue boxes show the peg task being performed to assess finger dexterity, and gray boxes show the simultaneous measurement of brain activation and spinal motor neuron excitability. (B) A schematic representation of the assessment of the neural basis with C4 × 3 (bilateral) and 3 × 5 probe fixation holders attached. The regions of interest are the left and right supplementary motor area (orange), primary motor area (red), orbitofrontal cortex (green), inferior frontal cortex (gray), frontal pole (blue), and dorsolateral prefrontal cortex (purple). F-waves were derived from the muscle group on the ball of the thumb by stimulating the median nerve at the hand joint, and the excitability of spinal motor neurons was evaluated by dividing F-wave by M wave amplitude. BP, baseline performance; Oxy-Hb, oxygenated hemoglobin.

The subject was positioned in a comfortable sitting posture using the backrest of a chair with a seat height of 410 mm and both forearms were placed on a desk with a height of 700 mm. First, finger dexterity was assessed using a Purdue pegboard as baseline performance (Pre-BP). Next, simultaneous measurement of brain activation and spinal motor neuron excitability was performed during 30 s of rest (Rest). The subject then performed AE and MIP for 30 s, respectively. This series of AEMIP intervention (AE followed by MIP) was repeated in six sets (AE1–6, MIP1–6), and brain activation and spinal motor neuron excitability were measured simultaneously in MIP1 and MIP6. In each MIP, the participants were instructed to recall a composite of information related to the motor task (information indirectly associated with muscular sensation) in addition to kinesthetic MIP (Fukumoto et al., 2022a). Finally, finger dexterity was reassessed using a Purdue pegboard (Post-BP; Fig. 1A).

### 2.5 Assessment of brain function

Near-infrared spectroscopy (NIRS; OT-R41; Hitachi, Ltd., Tokyo, Japan) was used to measure brain function with a sampling frequency of 10 Hz. Compared to total hemoglobin and deoxygenated hemoglobin, oxygenated hemoglobin (Oxy-Hb) is the most sensitive indicator of changes in regional cerebral blood flow (Hoshi et al., 2001, Strangman et al., 2003), so it was used to analyze changes in brain activation. NIRS measurements were taken during MIP1 and MIP6 in addition to the resting state, and the change of Oxy-Hb during MIP1 and MIP6 was calculated based on the resting state, and this was used as an index of changes in brain activation. The probe was attached to C4 × 3 (bilateral) and 3 × 5 probe fixation holders based on the international 10–20 method, and the regions of interest were the left and right SMA, M1, orbitofrontal cortex (OFC), inferior frontal cortex (IFC), frontal pole cortex (FPC), and dorsolateral prefrontal cortex (DLPFC) (Fukumoto et al., 2022b). Virtual registration was used to identify channel positions and brain regions (Tsuzuki et al., 2007). This method can estimate the brain region in which the channel is measured by converting the position of the virtual probe holder to the Montreal Neurological Institute standard brain coordinate system and projecting it onto the brain surface. The estimation error is within 13 mm, which is a practically acceptable level of spatial estimation accuracy.

There are two methods for analyzing data obtained from the brain: one is to focus on a single brain region and clarify the correspondence between brain structure and activation (activation level analysis of a single brain region), and the other is to assume that the brain is a single network and elucidate the brain network based on graph theory (network analysis) (Reijneveld et al., 2007). Here, both methods were employed because the understanding of functional changes in the brain during fast learning can be advanced by incorporating the viewpoint of the brain network in addition to focusing on a single brain region (Sami and Miall, 2013). First, in the activation analysis of a single brain region, the change in Oxy-Hb concentration in the region of interest (mM × mm) during each task (resting, MIP1, MIP6) for 30 s was calculated based on baseline fitting before and after the task and used as the level of activation. The average value of the channel data constituting each region of interest was used for each region of interest, and the analysis system software included in OT-R41 was used for NIRS data analysis processing. In network analysis, a network is represented by a node (a point) and an edge. In the case of a brain network, regions of interest correspond to nodes and functional connections correspond to edges. Therefore, each region of interest is considered a node, and the regions in which the correlation coefficient of the time-series data of 12 regions of interest obtained by NIRS data analysis exceeds a certain threshold are considered edges. In addition, “betweenness centrality,” an index that quantifies the influence of a node in connecting other nodes in the network, was employed; a higher value of betweenness centrality indicates that the node functions as an important region in information transmission (Freeman, 1978). In this study, the region with the highest betweenness centrality was defined as a hub (Fig. 1B).

### 2.6 Assessment of spinal motor neuron excitability

F-waves were used as an index for evaluating changes in the excitability of spinal motor neurons, which are complex muscle action potentials recorded in the corresponding muscles as retrograde impulses generated by maximal electrical stimulation of peripheral mixed nerve fibers travel up the axon and then down the axon as progressive impulses as a result of re-firing in anterior horn cells of the spinal cord. F-waves were recorded with a Viking Quest evoked electromyograph (ver. 20.11.1; Natus Medical, Inc., Pleasanton, CA, USA). F-wave recording conditions were as follows: the stimulating electrode was placed on the median nerve at the left wrist joint, the probe electrode was placed on the muscle group on the left thumb ball, the reference electrode was placed on the dorsal surface of the left first metacarpal head, and the ground electrode was placed on the palmar side of the left forearm. A bipolar bar electrode (Au) was used as the stimulating electrode, a 10-mm diameter electroencephalography cup electrode (Ag/AgCl) as the reference/probe electrode, and a 30-mm diameter plate ground electrode (stainless) as the ground electrode (Fig. 1C). The F-wave analysis item was the amplitude F/M ratio, which reflects the increase in the number of muscle fibers that re-fire in the anterior horn cells of the spinal cord (Eisen and Odusote, 1979). F-waves were measured during MIP1 and MIP6 in addition to the resting state. On the basis of the F/M ratio, the amplitude F/M ratio change during MIP1 and MIP6 in relation to rest was calculated, and this was used as an index of spinal motor neuron excitability (Fig. 1B).

### 2.7 Assessment of finger dexterity

The Purdue pegboard is performed worldwide and evaluation reference values exist. Although the basic method of operation uses both hands, reference values have been established for operations using only the non-dominant hand (Seol et al., 2023, Park and Son, 2022). The specific task procedure is to take one pin from the left-most receiving plate with the left hand and insert the pin into the first empty hole at the top of the left row. The procedure is repeated for 30 s and the number of pins that could be inserted at the end of that period was used as the score. In this study, all participants were right-handed, so only the left hand was used.

### 2.8 Determination of the introspective state of the participants

To determine the clarity of MIP, we conducted an original evaluation using a modified version of mental chronometry (Guillot et al., 2012). First, the number of pegs the participants could move during each MIP (MIP1–6) was stated during the 30-s MIP. These values were compared in with the actual number of pegs that were moved in each AE (AE1–6).

Next, the participants were instructed to perform MIP in a kinesthetic manner, but the composite sensation included the movement of the pegs. To determine whether the focal point of this combined recall information was a muscular sensory association (e.g., the phase to pick up a peg from the plate, the phase to hold and move the peg, and the phase to insert the peg into the hole) or just a visual image (e.g., a visual scene of another person moving the pegs), the participants were asked to state the focus of the recalled information in each MIP.

### 2.9 Data analysis

The purpose of this study was to determine whether central nervous system activity during MIP differs according to Digit span scores, which estimate phonological loops. For the Normal score of Digit Span: Backward, 74 % of participants scored between 4 and 6 digits, and when limited to younger participants, the median was 6 digits (Woods et al., 2011). Furthermore, those who scored above 7 digits were reported to have superior attention and WM compared to those who scored 6 digits or below (Zenisek et al., 2016). Therefore, for WM function grouping, subjects with a score of 5–6 digits in Digit Span: Backward were included in the Normal WM group, while those with a score of ≥ 7 digits were included in the Good WM group. GRETNA with MATLAB R2020a (The MathWorks, Inc., Natick, MA, USA) was used to calculate betweenness centrality based on graph theory network analysis. In this study, multiple binary networks were created by systematically shifting the thresholds, and graph-theoretic indices were calculated for these networks and averaged across thresholds. Specifically, a correlation matrix based on the correlation coefficients between regions for 30 s (300 points) of time-series data during the task obtained from the activation analysis of each brain region was created. Next, the threshold value from the top 10 % to the top 30 % of the coupling strength was shifted in 1 % increments, and a binarized network was created at each threshold value. Finally, the graph theory indices for the networks binarized at each threshold were averaged (Wang et al., 2015). In the Shapiro-Wilk test, the Oxy-Hb change, amplitude F/M ratio change, and peg score in MIP1 and MIP6 did not show normality in any of the regions with the highest values of betweenness centrality. First, to confirm that there were no differences in physical factors between the two groups, the Peg scores at Pre-BP were compared between the two groups using the Mann-Whitney *U* test. Next, the Oxy-Hb change, amplitude F/M ratio change, and betweenness centrality were calculated for both groups (Normal WM group, Good WM group) and over time (MIP1, MIP6), and the peg score was calculated for both groups (Normal WM group, Good WM group) and time course (Pre-BP, Post-BP). In the case of an interaction, a simple main effect test was performed, and in the case of no interaction, a main effect test was performed. An r value was calculated based on the Z value. In addition, to confirm the correlation between WM and neural activity levels at the individual level during MIP, we evaluated the monotonic relationship (ordinal relationship) between two variables (Digit Span scores and Oxy-Hb change and amplitude F/M ratio change) using Spearman's correlation analysis. The significance level was set at less than 5 % in both cases, and SPSS (ver. 26.0; IBM, Inc., Armonk, NY, USA) was used as the statistical analysis software.

## 3 Results

### 3.1 Attributes of the subjects

One subject with a Digit Span: Backward cutoff score of ≤ 4 digits was excluded, and 20 subjects (10 males/10 females/mean age 20.5 ± 1.0 years) were finally included in the study. Thirteen subjects (five males/eight females, mean age 20.5 ± 1.2 years) were included in the Normal WM group (Digit Span: Backward 5–6 digits) and seven subjects (four males/three females, mean age 20.3 ± 0.7 years) were included in the Good WM group (Digit Span: Backward ≥ 7 digits). All subjects used the strategy of muscular-sensory imagery for MIP in 97 % (116/120) of all trials. Furthermore, for kinesthetic imagery, 57 % (68/120) of the trials focused attention on the phase of picking up the peg from the plate, 13 % (15/120) on the phase of holding and moving the peg, and 27 % (33/120) on the phase of inserting the peg into the hole. Details of each subject’s WM function and the focus of attention during MIP are shown in Table 1.

**Table 1:** Focus of the subjects’ working memory function and recall in each motor imagery practice (MIP).

**Participant**	**Digit Span**	**Pre-BP**	**MIP 1**	**MIP 2**	**MIP 3**	**MIP 4**	**MIP 5**	**MIP 6**	**Participant**	**Digit Span**	**Pre-BP**	**MIP 1**	**MIP 2**	**MIP 3**	**MIP 4**	**MIP 5**	**MIP 6**
A	5	13	on the move	on the move	take out from a dish	put a pin in a hole	put a pin in a hole	put a pin in a hole	N	7	11	take out from a dish	take out from a dish	take out from a dish	take out from a dish	take out from a dish	take out from a dish
B	5	13	on the move	take out from a dish	take out from a dish	take out from a dish	take out from a dish	take out from a dish	O	7	13	take out from a dish	Scene with Peg going fast	Scene with Peg going accuracy	put a pin in a hole	put a pin in a hole	take out from a dish
C	5	13	take out from a dish	on the move	Scene with no attention take the pin	put a pin in a hole	on the move	put a pin in a hole	P	7	12	take out from a dish	take out from a dish	take out from a dish	put a pin in a hole	take out from a dish	take out from a dish
D	6	11	on the move	on the move	take out from a dish	put a pin in a hole	on the move	put a pin in a hole	Q	7	13	take out from a dish	take out from a dish	take out from a dish	take out from a dish put a pin in a hole	take out from a dish put a pin in a hole	take out from a dish put a pin in a hole
E	6	13	on the move	take out from a dish	take out from a dish	take out from a dish	on the move	take out from a dish	R	8	13	put a pin in a hole	take out from a dish	put a pin in a hole	take out from a dish	take out from a dish	take out from a dish
F	6	13	put a pin in a hole	put a pin in a hole	take out from a dish on the move	put a pin in a hole	put a pin in a hole	put a pin in a hole	S	8	13	on the move	take out from a dish	take out from a dish	take out from a dish	put a pin in a hole	take out from a dish
G	6	14	on the move	put a pin in a hole	take out from a dish	take out from a dish	take out from a dish	take out from a dish	T	8	15	take out from a dish	put a pin in a hole	on the move	on the move	take out from a dish	take out from a dish
H	6	13	take out from a dish	take out from a dish	take out from a dish	Scene of peg doing motion	take out from a dish	take out from a dish									
I	6	14	take out from a dish	put a pin in a hole	put a pin in a hole	put a pin in a hole	put a pin in a hole	put a pin in a hole									
J	6	10	take out from a dish put a pin in a hole	take out from a dish put a pin in a hole	take out from a dish put a pin in a hole	take out from a dish on the move	take out from a dish put a pin in a hole	take out from a dish on the move									
K	6	12	on the move	take out from a dish	put a pin in a hole	take out from a dish	on the move	take out from a dish									
L	6	18	take out from a dish	take out from a dish	put a pin in a hole	take out from a dish	put a pin in a hole	take out from a dish									
M	6	12	put a pin in a hole	take out from a dish	take out from a dish	take out from a dish	put a pin in a hole	put a pin in a hole									

Digit Span: Backward measures working memory function in terms of the number of digits that can be recited backwards, with a minimum of two digits and a maximum of nine digits, but in this study, five or more digits were used.

Next, the number of pegs that could be moved during MIP and AE with motor execution was compared in a way that corresponded to the number of pegs that could be moved as assumed during MIP, and we found that both groups overestimated the number of pegs they imagined they could move during MIP, but this dissociation was more pronounced in the Normal WM group (Table 2).

**Table 2:** Comparison of peg scores between actual execution (AE) and motor imagery practice (MIP).

	**Normal WM Group**			**Good WM Group**		
	**Peg score(MIP)**	**Peg score(AE)**	***p*****-value**	**Peg score(MIP)**	**Peg score(AE)**	***p*****-value**
AE1-MIP1	15 (13−15)	14 (13−15)	0.720	14 (14–14.5)	14 (12.5–14.5)	0.498
AE2-MIP2	17 (15−18)	14 (13−15)	0.009	14 (14−15)	14 (13.5–14)	0.197
AE3-MIP3	17 (16−19)	14 (13−16)	0.008	16 (14−16)	14 (14–14.5)	0.102
AE4-MIP4	19 (16−20)	15 (14−16)	0.016	16 (15–16.5)	15 (14−15)	0.086
AE5-MIP5	19 (16−20)	15 (14−16)	0.013	16 (15.5–17)	15 (14–15.5)	0.144
AE6-MIP6	17 (16−21)	15 (14−16)	0.007	16 (15.5–16.5)	15 (14.5–16)	0.129

WM, working memory.

### 3.2 Activation in single brain regions

**Fig. 2:**
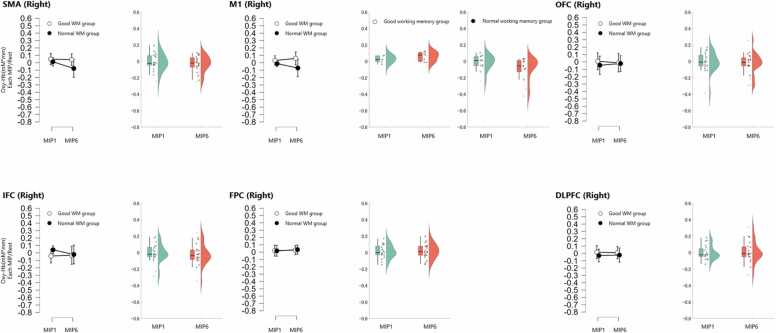
•**SMA**: F(1, 18) = 0.155, *p* = 0.698, η²ₚ = 0.009 MIP1 vs. MIP6: *p* = 0.370, *r* = 0.20•**OFC**: F(1, 18) = 0.000, *p* = 0.994, η²ₚ = 0.000 MIP1 vs. MIP6: *p* = 0.391, *r* = 0.19•**IFC**: F(1, 18) = 0.506, *p* = 0.486, η²ₚ = 0.027 MIP1 vs. MIP6: *p* = 0.263, *r* = 0.25•**FPC**: F(1, 18) = 0.000, *p* = 0.998, η²ₚ = 0.000 MIP1 vs. MIP6: *p* = 0.911, *r* = 0.03•**DLPFC**: F(1, 18) = 0.170, *p* = 0.685, η²ₚ = 0.009 MIP1 vs. MIP6: *p* = 1.000, *r* = 0.00Single brain region activation analysis of the right hemisphere. Oxy-Hb changes were analyzed across six right-hemisphere ROIs (SMA, M1, OFC, IFC, FPC, DLPFC). For M1, a two-way ANOVA with group (Good vs. Normal working memory; WM) and phase (MIP1 vs. MIP6) revealed a significant interaction between group and phase (F(1, 18) = 6.478, *p* = 0.020, η²ₚ = 0.265). Based on this interaction, simple main effect analyses were conducted within each group. A significant decrease in Oxy-Hb was found from MIP1 to MIP6 in the Normal WM group (*p* = 0.009, *r* = 0.73), whereas no significant change was observed in the Good WM group (*p* = 0.237, *r* = 0.45).In contrast, for the other ROIs (SMA, OFC, IFC, FPC, DLPFC), no significant main effects or interactions were observed. Therefore, comparisons between MIP1 and MIP6 were conducted across all participants (not separated by group). No significant changes were found in any of the following regions: Violin plots display the distribution of individual Oxy-Hb changes by group (M1) or across the entire sample (other ROIs).

In the simple main effect test, there was an interaction effect for M1 and a decrease in activation in MIP6 compared to MIP1 only in the Normal WM group, and there was no interaction effect for the SMA, OFC, IFC, FPC, and DLPFC, and no difference in the main effect test (Fig. 2; Table 3).

**Table 3:** Measured changes in brain activation and spinal motor neuron excitability and motor skills.

**Brain function**		**Normal WM Group**		**Good WM Group**	
Brain region: Oxy-Hb(mM*mm)	**Hemisphere**	**MIP1/Rest**	**MIP6/Rest**	**MIP1/Rest**	**MIP6/Rest**
The supplementary motor area	Right	−0.04 (−0.09 - −0.01)	−0.06 (−0.13 - −0.02)	0.05 (−0.02–0.09)	0.06 (0.02–0.08)
	Left	0.04 (−0.03–0.09)	−0.05 (−0.10 - −0.02)	0.04 (0.01–0.11)	0.02 (−0.01–0.04)
The primary motor area	Right	0.01 (−0.05–0.06)	−0.06 (−0.09–0.01)	0.02 (0.01–0.07)	0.08 (0.01–0.10)
	Left	0.01 (−0.04–0.03)	−0.01 (−0.06–0.01)	0.03 (0.00–0.09)	0.04 (0.01–0.06)
The orbitofrontal cortex	Right	−0.01 (−0.05–0.06)	−0.05 (−0.05–0.01)	−0.01 (−0.04–0.07)	0.03 (−0.02–0.07)
	Left	−0.05 (−0.10 - −0.01)	−0.04 (−0.16–0.03)	−0.03 (−0.03–0.05)	0.05 (−0.04–0.08)
The inferior frontal cortex	Right	0.02 (−0.03–0.07)	−0.05 (−0.08–0.02)	−0.05 (−0.08 - −0.02)	−0.01 (−0.08–0.07)
	Left	−0.01 (−0.07–0.03)	−0.03 (−0.10–0.06)	−0.04 (−0.05–0.00)	0.00 (−0.03–0.08)
The frontopolar cortex	Right	−0.01 (−0.06–0.09)	0.00 (−0.03–0.08)	0.02 (−0.01–0.04)	0.03 (0.01–0.07)
	Left	0.00 (−0.07–0.02)	−0.02 (−0.04–0.03)	0.01 (0.00–0.05)	0.06 (0.04–0.08)
The dorsolateral prefrontal cortex	Right	−0.03 (−0.08–0.03)	−0.02 (−0.05–0.02)	−0.02 (−0.03–0.05)	−0.01 (−0.01–0.07)
	Left	0.04 (0.00–0.07)	0.01 (−0.04–0.06)	0.05 (0.00–0.08)	0.03 (−0.02–0.07)
**Brain region: Average Betweenness Centrality**		**MIP1/Rest**	**MIP6/Rest**	**MIP1/Rest**	**MIP6/Rest**
The supplementary motor area	Right	0.00 (0.00–0.07)	0.00 (0.00–0.43)	0.39 (0.00–1.55)	0.00 (0.00–0.19)
	Left	0.00 (0.00–0.00)	0.00 (0.00–0.00)	0.00 (0.00–0.06)	0.00 (0.00–0.00)
The primary motor area	Right	0.00 (0.00–0.03)	0.00 (0.00–0.00)	0.00 (0.00–0.00)	0.00 (0.00–0.00)
	Left	0.00 (0.00–0.00)	0.00 (0.00–0.00)	0.00 (0.00–0.95)	0.00 (0.00–0.00)
The orbitofrontal cortex	Right	0.45 (0.00–2.86)	1.68 (0.64–2.04)	0.27 (0.14–0.61)	0.88 (0.05–2.61)
	Left	1.14 (0.02–3.67)	0.31 (0.00–2.23)	0.23 (0.00–0.74)	1.67 (0.42–2.56)
The inferior frontal cortex	Right	0.29 (0.00–1.49)	0.19 (0.00–1.54)	1.33 (0.00–1.64)	0.07 (0.04–2.15)
	Left	0.30 (0.00–2.44)	0.14 (0.00–0.93)	2.01 (0.00–6.45)	0.66 (0.00–1.57)
The frontopolar cortex	Right	2.24 (1.76–4.98)	0.24 (0.03–1.71)	1.62 (0.17–2.98)	0.47 (0.14–1.20)
	Left	0.14 (0.00–1.46)	0.00 (0.00–0.57)	1.72 (1.47–3.12)	1.03 (0.44–2.00)
The dorsolateral prefrontal cortex	Right	1.44 (0.00–1.67)	0.38 (0.00–1.37)	0.00 (0.00–4.37)	0.75 (0.00–2.80)
	Left	1.36 (0.48–1.61)	0.72 (0.05–1.71)	0.08 (0.02–2.04)	0.73 (0.34–1.18)
**Spinal motor nerve function**		**MIP1/Rest**	**MIP6/Rest**	**MIP1/Rest**	**MIP6/Rest**
F/M amplitude ratio (%)	Left	0.22 (0.11–0.41)	0.31 (0.05–0.83)	0.48 (0.20–1.09)	0.05 (−0.38–0.14)
**Fingertip dexterity**		**Pre-BP**	**Post-BP**	**Pre-BP**	**Post-BP**
Peg score (number)	Left	13.00 (12.00–13.00)	16.00 (15.00–17.00)	13.00 (12.50–13.00)	16.00 (15.00–16.50)

The values are presented as medians (first quartile – third quartile). BP, baseline performance; MIP, motor imagery performance; Oxy-Hb, oxygenated hemoglobin.

**Fig. 3:**
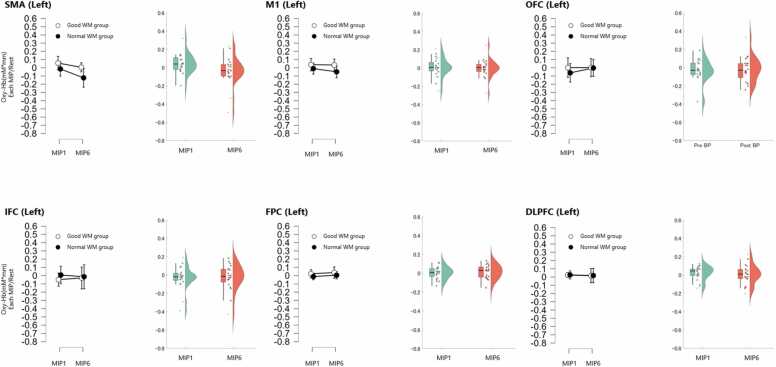
•**SMA**: F(1, 18) = 0.354, *p* = 0.559, η²ₚ = 0.019 MIP1 vs. MIP6: *p* = 0.021, *r* = 0.52•**M1**: F(1, 18) = 0.073, *p* = 0.791, η²ₚ = 0.004 MIP1 vs. MIP6: *p* = 0.108, *r* = 0.36•**OFC**: F(1, 18) = 0.497, *p* = 0.490, η²ₚ = 0.027 MIP1 vs. MIP6: *p* = 0.370, *r* = 0.20•**IFC**: F(1, 18) = 0.117, *p* = 0.737, η²ₚ = 0.006 MIP1 vs. MIP6: *p* = 0.765, *r* = 0.07•**FPC**: F(1, 18) = 0.028, *p* = 0.869, η²ₚ = 0.002 MIP1 vs. MIP6: *p* = 0.073, *r* = 0.40•**DLPFC**: F(1, 18) = 0.091, *p* = 0.766, η²ₚ = 0.005 MIP1 vs. MIP6: *p* = 0.313, *r* = 0.23Single brain region activation analysis in the left brain. Oxy-Hb changes were analyzed across six left-hemisphere ROIs (SMA, M1, OFC, IFC, FPC, DLPFC). No significant group × phase interactions or main effects of group were observed in any region. Therefore, comparisons between MIP1 and MIP6 were conducted across all participants. A significant decrease in Oxy-Hb was found in SMA (*p* = 0.021, *r* = 0.52), while no significant differences were observed in the other ROIs. The results are as follows: Violin plots illustrate the distributions of Oxy-Hb changes between MIP1 and MIP6 for each ROI.

There was no interaction between the SMA, M1, OFC, IFC, FPC, and DLPFC as changes in left brain activation. In the main effect test, a decrease in activation was observed only in the SMA in MIP6 compared to MIP1 (Fig. 3; Table 3).

### 3.3 Brain network analysis

**Fig. 4:**
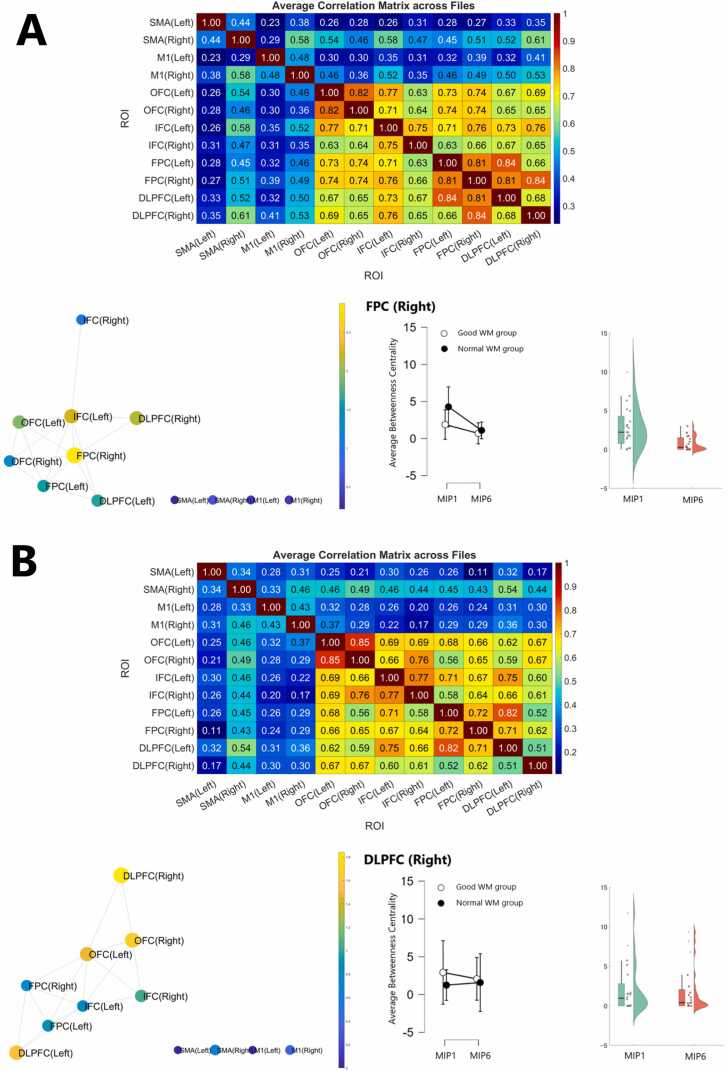
Network formation in the brain in terms of functional connectivity.(A) Network analysis in MIP1. The upper panel displays the cross-correlation matrix among regions of interest (ROIs). The lower left shows the network diagram based on top-ranked correlation coefficients. FPC (Right) exhibited the highest Betweenness Centrality. A generalized linear mixed model (GLMM) was used to perform a two-way ANOVA (group × phase) on Betweenness Centrality in FPC (Right). No significant interaction was observed (F(1, 18) = 1.829, *p* = 0.193, η²ₚ = 0.280); however, a significant decrease in centrality was found in MIP6 compared to MIP1 across both groups (*p* < 0.001, *r* = 0.77). (B) Network analysis in MIP6. The upper panel again shows the correlation matrix; the lower left shows the corresponding network structure. DLPFC (Right) exhibited the highest Betweenness Centrality in MIP6. The same GLMM-based analysis for DLPFC (Right) revealed no significant interaction (F(1, 18) = 0.205, *p* = 0.656, η²ₚ = 0.036) and no significant difference between MIP1 and MIP6 (*p* = 0.501, *r* = 0.15).

The mean betweenness centrality of each region of interest at all thresholds was calculated based on the binarization of the upper correlation coefficients between regions. The results showed that the bilateral SMA and M1 were not included in the network, and that the FPC (right) for MIP1 and DLPFC (right) for MIP6 showed higher betweenness centrality. The FPC (right) showed no interactions, but it was lower in MIP6 compared to MIP1 in the main effect test, and the DLPFC (right) showed no interactions and no differences in the main effect test (Fig. 4A, B; Table 3).

### 3.4 Changes in spinal motor neuron excitability

**Fig. 5:**
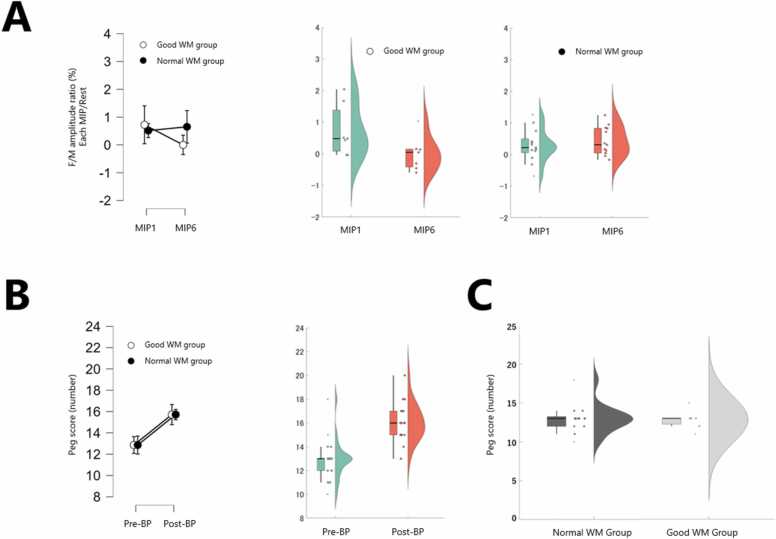
Spinal motor neuron excitability and finger dexterity. (A)Spinal motor neuron excitability was assessed using the change in amplitude F/M ratio from rest during each motor imagery practice (MIP) session. The unit is percentage (%), but it represents a change in value, not a rate. A significant interaction between group and phase was observed (F(1, 18) = 10.282, p = 0.005, η²ₚ = 0.364). Simple main effect analysis revealed a significant decrease in the Good Working Memory (WM) group from MIP1 to MIP6 (p = 0.046, r = 0.71), whereas no significant change was observed in the Normal WM group (p = 0.295, r = 0.30). (B) Manual dexterity was evaluated based on the peg score (number of pegs inserted in 30 s). No significant interaction was found (F(1, 18) = 0.367, p = 0.552, η²ₚ = 0.020), but a significant main effect of phase indicated improvement from MIP1 (Pre-BP) to MIP6 (Post-BP) (p < 0.001, r = 0.88). (C) A baseline comparison of peg scores (Pre-BP) between groups revealed no significant difference (p = 0.966, r = 0.01), indicating similar initial manual dexterity between the Good and Normal WM groups.

An interaction was found in the amplitude F/M ratio, and a simple main effect test showed a decrease in spinal motor neuron excitability in MIP6 compared to MIP1 only in the Good WM group (Fig. 5A; Table 3).

### 3.5 Finger dexterity

No interaction was found in the peg score, but an improvement in finger dexterity was found in Post-BP compared to Pre-BP in the main effect test (Fig. 5B; Table 3). In addition, no statistical differences were observed in the Pre-BP scores between the two groups (Fig. 5C).

### 3.6 Correlation between cognitive function and neural activity patterns

**Fig. 6:**
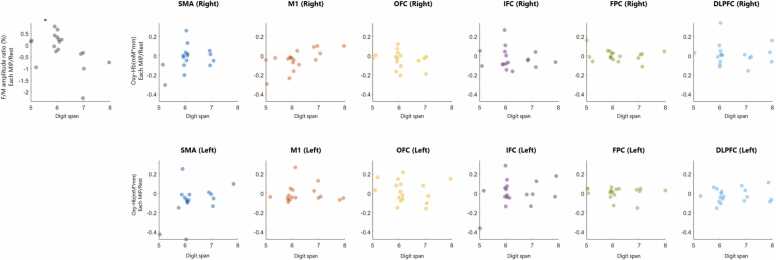
Correlation between cognitive function and neural activity patterns The relationship between Digit span scores and neural response changes during motor imagery practice (MIP) was analyzed using Spearman’s rank correlation coefficient (*rₛ*). Neural indices included spinal motor neuron excitability (amplitude F/M ratio change) and Oxy-Hb changes in each cortical ROI. Spinal motor neuron excitability (amplitude F/M ratio change) showed a significant negative correlation with Digit span (*rₛ* = –0.47, *p* = 0.035), suggesting that individuals with higher working memory span exhibited greater reductions in excitability. Additionally, a significant positive correlation was observed in M1 (Right) (*rₛ* = 0.48, *p* = 0.031). No significant correlations were found in the other ROIs:•**SMA (Right)**: *rₛ* = 0.28, *p* = 0.229•**OFC (Right)**: *rₛ* = –0.02, *p* = 0.922•**IFC (Right)**: *rₛ* = 0.29, *p* = 0.211•**FPC (Right)**: *rₛ* = 0.03, *p* = 0.914•**DLPFC (Right)**: *rₛ* = –0.02, *p* = 0.919•**SMA (Left)**: *rₛ* = 0.16, *p* = 0.513•**M1 (Left)**: *rₛ* = 0.10, *p* = 0.675•**OFC (Left)**: *rₛ* = –0.15, *p* = 0.535•**IFC (Left)**: *rₛ* = 0.15, *p* = 0.529•**FPC (Left)**: *rₛ* = 0.00, *p* = 0.989•**DLPFC (Left)**: *rₛ* = –0.12, *p* = 0.603

A significant positive correlation was observed between Digit Span scores and right M1 Oxy-Hb change, but no significant correlations were observed in other left and right brain regions. Furthermore, amplitude F/M ratio change showed a significant negative correlation with Digit Span scores (Fig. 6).

## 4 Discussion

Of the total MIP attempts by all subjects, 97 % were performed using kinesthetic imagery, and MIP was associated with an overestimated recall compared to actual ability in AE, which was more pronounced in the Normal WM group. Both groups showed an improvement in finger dexterity after AEMIP. However, there were differences in the changes of the neural basis, with a decrease in SMA (left) activation in both groups at MIP6 compared to MIP1, but also a decrease in the excitability of spinal motor neurons at MIP6 compared to MIP1 in the Good WM group, and a decrease in M1 (Right) activation in the Normal WM group. Similarly, as the WM score (Digit span) increased, activation of the right M1 was observed, and at the same time, the excitability of spinal motor neurons decreased. Furthermore, network analysis showed that the bilateral SMA and M1 were independent of each other. The region with the highest betweenness centrality in the network was the FPC (right) in MIP1, which shifted to the DLPFC (right) in MIP6. In MIP6, the FPC (right) showed a lower value, while the betweenness centrality of the DLPFC (right) did not change compared to MIP1.

### 4.1 Motor skill changes with AEMIP

The learning forms involved in motor skill improvement include “error-based learning,” “reinforcement learning,” “use-dependent plasticity,” and “cognitive strategies,” and although their relative contributions vary depending on the motor task, they are not completely separate and are believed to achieve skill acquisition by their interrelatedness (Spampinato and Celnik, 2021). These forms of learning are considered to be brought about by AE, but recently it has become clear that MIP, which is cognitive activity, can promote this learning (Kim et al., 2022, Ruffino et al., 2019). We have previously shown that the use of AEMIP can achieve higher motor skill level acquisition than AE alone when MIP strategies are taken into account (Fukumoto et al., 2024a). In other words, since AEMIP had MIP inserted in the intertrial interval, it likely enhanced the effect of MIP alone or the effect of fast learning in the form of facilitating the appropriate modification of the exercise program during this period.

However, given the requirement for WM to generate MIP, individual differences in the effects of MIP may be influenced by the WM function available to each individual, and this was examined in this study. Activity in frontal lobe regions, mainly in the DLPFC, is observed during motor tasks before learning (Seidler et al., 2012) and during MIP (Fukumoto et al., 2022b), which supports the strong need for WM in the early stages of learning and in obtaining MIP effects. However, the WM functions assessed by Digit Span as a pool of linguistic information and is therefore unlikely to be considered as memory that serves as material for MIP generation. In other words, the phonological loop can be interpreted as a function related to the “process” of processing linguistic instructions, efficiently retaining and utilizing that information to generate and maintain MI in MIP executed under linguistic instructions, and the individual differences in its capacity and ability influence the effects of MIP (Fukumoto et al., 2024b). In this previous study, which examined the effects of MIP alone, despite MIP being administered alone and only once, individuals with a Digit Span of four digits or fewer received only a small benefit from MIP in terms of improvements in finger dexterity compared to those with a Digit Span of five digits or more. Therefore, even if MIP is repeated, it is unlikely that equivalent effects can be expected for individuals with a Digit Span of four digits or fewer. Therefore, as adopted in this study, when subjects possess a certain level of WM function (≥5 digits), they are likely to be able to perform a certain clear MIP, and there may have been no difference in the changes in motor skills as an effect of MIP.

However, caution is also required when considering the factors that led to similar improvements in finger dexterity between the two groups through MIP. As differences in physical factors directly contributing to the learning process, it is already known that initial motor skill differences can influence the MIP effect (Mao et al., 2022, Dahm and Rieger, 2019, Kraeutner et al., 2018). However, in this study, no differences in initial finger dexterity skills were observed between the two groups, thereby alleviating concerns regarding this point. Therefore, the question arises whether differences in WM affect the MIP effect. In the evaluation of clarity in MIP, which is an improved version of mental chronometry, the Normal WM group exhibited significantly overestimated skill during MIP compared to AE, indicating that the Normal WM group gradually loses clarity when executing repeated MIP. However, the fact that improvements in finger dexterity through MIP were similar between the two groups suggests that even if WM influences the clarity of MIP, it does not necessarily have a negative impact on motor learning ability. This seemingly contradictory result is thought to stem from the multifaceted nature of MIP. Clarity of MIP does not necessarily explain changes in motor skills mediated by MIP (Ruffino et al., 2017), and MIP may be influenced by psychological factors such as self-efficacy, motivation, and mood, in addition to WM (Schuster et al., 2011). Therefore, even if MIP clarity is low, if WM is maintained at a certain level (e.g., five digits or more in digit span), the MIP effect may be compensated by intentionally designed MIP strategies (Fukumoto et al., 2022a) or high motivation and self-efficacy.

### 4.2 Modulation of the neural basis by AEMIP

In activation analysis of single brain regions, no change was observed between MIP1 and MIP6 in the frontal lobe region, where cognitive activity occurs, but network formation was confirmed in brain network analysis based on graph theory. The highest betweenness centrality was observed in the right FPC in MIP1 and the right DLPFC in MIP6, but only the betweenness centrality of the FPC was lower in MIP6 than in MIP1. Since the FPC is considered to be involved in reevaluating decisions made in the past (metacognition) (Okuda et al., 2003, Tsujimoto et al., 2010), this result may reflect the fact that MIP1 in particular often reflected the subjects’ motor actions during MIP. The DLPFC is a core area of WM that is specifically and additionally active in MIP, where internal simulation continues offline (Vry et al., 2012). The betweenness centrality of the DLPFC did not decrease in MIP6 compared to MIP1, suggesting that the DLPFC was consistently necessary for the generation of MIP. Conversely, these brain networks did not include motor-related regions such as the SMA and M1. This suggests that the lateral prefrontal cortex, which is responsible for cognitive activity, and motor-related regions may activate networks during MIP. It has been reported that the WM network in the lateral prefrontal regions, mainly in the DLPFC, is responsible for WM function, which performs operations while retaining information, and executive functions such as planning, attention allocation, and inhibition (Baddeley, 2000), but not the motor-related regions, especially the M1 (Meunier et al., 2009). From the above, it seems that the above network formation indicated the quantitative aspect of the availability of MIP execution, in that MIP represents the activation of WM.

The SMA and M1, which were not included in the WM network, may have been involved in the qualitative aspects of MIP, i.e., how MIP is performed. In the present study, the right M1 was less active in the Normal WM group at MIP6 compared to MIP1, and the excitability of spinal motor neurons was reduced in the Good WM group at MIP6 compared to MIP1. Furthermore, as a similar feature, in the correlation analysis between WM and neural activity at each level, when WM was high, changes in activation in right M1 were also high, and excitability changes in spinal motor neurons were low. M1 activation is reportedly observed when MIP is performed well (Dechent et al., 2004) and there is no increase in the excitability of spinal motor neurons when MIP is performed clearly (Nomura et al., 2017). Thus, these regions are related to the qualitative aspect of MIP, and the change in the excitability of spinal motor neurons may more sensitively reflect a slight difference in MIP when WM function is higher and clear MIP is performed, in particular. The clarity of MIP can be estimated by using a modified version of mental chronometry. First, the Good and Normal WM groups both overestimated their skills during MIP compared to during AE, but this is not a problem because it is an important issue when attempting to improve skills through cognitive activities such as MIP and exercise observation (Ikegami and Ganesh, 2014). The problem is that there was no significant differences in AE and MIP deviations in the Good WM group, while the Normal WM group showed significant differences in all remaining trials except for the AE1-MIP1 comparison. This may be due to the characteristics of the Normal WM group, such as mental fatigue that occurred during repeated MIP, which reduced the clarity of MIP, and as a result, the Normal WM group became less confident when estimating their ability. When the Normal WM group was unable to perform clear MIP for a certain period, it was difficult for them to say that MIP had been performed well, and this was reflected in a decrease in the amount of M1 activation.

Although M1 activity and the changes in spinal motor neuron excitability seem to capture the qualitative aspects of MIP, discrepancies were observed in the increase or decrease in activity in each group. Until now, excitatory changes in spinal motor neurons during MIP have been thought to be influenced via descending fibers in response to changes in the activity of the superior centers (Suzuki et al., 2014). However, there have been reports of discrepancies between the excitability of corticospinal tracts and spinal motor neuron function during MIP (Aoyama et al., 2019), and it remains to be examined whether the same behavior can occur at the spinal cord level following changes in central brain regions, especially in the M1. In addition, the SMA is another area other than the M1 that influences changes in spinal motor neuron excitability. Although SMA activity during finger dexterity movements is thought to play the main role in generating motor programs and projecting to the M1 (Takakusaki, 2013), the SMA is said to have fibers that also project directly to the spinal cord (Luppino and Rizzolatti, 2000). Furthermore, the SMA affects the excitability of spinal motor neurons ipsilaterally by descending fibers through the brainstem reticular formation (Keizer and Kuypers, 1989). In the present study, left SMA activity was decreased in MIP6 compared to MIP1, consistent with changes in the excitability of spinal motor neurons in the Good WM group. Therefore, it is possible that the changes in spinal motor neuron excitability were influenced by ipsilateral descending fibers. However, no interaction between the groups was observed, so we need to examine the factors that determine the changes of spinal motor neuron excitability, including the effects of brain regions other than the central brain region (motor-related region).

### 4.3 Limitations

One of the main limitations of this study is that it evaluated WM function only using the Digit Span: Backward test and only looked at one aspect of WM as a whole. In addition, NIRS is useful for the simultaneous measurement of brain activation in parallel with changes in the excitability of spinal motor neurons using evoked electromyography. However, because NIRS does not have high spatial resolution due to the characteristics of its equipment, it is not possible to observe the brain regions (hand regions) matched to the target muscles for the evaluation of changes in spinal motor neuron excitability. Furthermore, in the evaluation of finger dexterity, the difficulty of setting thresholds for behavioral assessment may have been a contributing factor. Even in the same peg task, when the task was set at a high level of difficulty, such as in the Grooved Pegboard Test, it cannot be ruled out that differences in WM may have caused changes in finger dexterity based on MIP.

## 5 Conclusions

First, as a common feature regardless of the WM, AEMIP produced the same degree of motor skill improvement when WM function was above the cutoff value. The WM network centered on the DLPFC was identified in repeated MIP during the motor skill improvement process, but the motor-related regions did not belong to this network. The WM network seems to be involved in quantitative aspects such as the generation of MIP. Next, as the central neural basis for the different activity patterns of different WMs, the repeated execution of MIP during motor skill improvement showed the decreased excitability of spinal motor neurons when WM function was high, and decreased M1 activity along with decreased MIP clarity when WM function was normal. These observations were consistent with changes in the excitability of central brain regions (motor-related regions) and spinal motor neurons in terms of MIP clarity.

## Compliance with ethical standards

This study adhered to the principles of the Declaration of Helsinki and was approved by the Ethics Committee of the Kansai University of Health Sciences (approval number: 23–20). All participants signed an informed consent statement prior to participation in the study.

## Funding

This research was supported by a Research Grant for Physiotherapy from the Japanese Society of Physical Therapy in 2023 (JSPT23–007).

## CRediT authorship contribution statement

**Yuki Fukumoto:** Writing – review & editing, Writing – original draft, Supervision, Project administration, Methodology, Investigation, Funding acquisition, Formal analysis, Conceptualization. **Hiroki Bizen:** Writing – review & editing, Validation, Methodology, Investigation. **Marina Todo:** Writing – review & editing, Validation, Methodology, Investigation. **Naoki Yoshida:** Writing – review & editing, Validation, Methodology, Formal analysis. **Toshiaki Suzuki:** Writing – review & editing, Validation, Methodology, Formal analysis.

## Declaration of Competing Interest

The authors declare that they have no known competing financial interests or personal relationships that could have appeared to influence the work reported in this paper.
